# Finding dolomitic melnoite diatreme at Badou in the Laiwu–Zibo area, Shandong province, China

**Published:** 2004-06-01

**Authors:** Akiko Goto, Hirokazu Fujimaki, Toshiro Morikiyo, Jianming Liu

**Affiliations:** *) Institute of Mineralogy, Petrology and Economic Geology, Tohoku University, Aramaki aza Aoba, Aoba-ku, Sendai, Miyagi 980-8578, Japan; **) Department of Geology, Shinshu University, 3-1-1, Asahi, Matumoto, Nagano 390-8621, Japan; ***) Research Center of Mineral Resources Exploration Chinese Academy of Sciences, P. O. Box 9701, 100101 Beijing, China

**Keywords:** Melnoite, ultramafic lamprophyre, carbonatite, melnoite-carbonatite-glimmerite association, Badou, Laiwu-Zibo area

## Abstract

We found a highly dolomitic lamprophyre diatreme at Badou, Shandong province, China for the first time. Although a number of silicate-rich carbonatite and glimmerite dikes and sheets were found around Badou, no alkaline rock–carbonatite plutonic complex exists nearby. Dolomitic lamprophyre diatreme that is not related with plutonic complex is not known yet either, and this is the first report of such an unusual occurrence. We report the whole-rock major and trace element chemistries of the lamprophyric rocks. The rocks consist of dominant dolomite with associated phlogopite, clinopyroxene, olivine, apatite and carry a number of xenoliths. Judging from all the geochemical and mineralogical data, the rock from Badou pipe should belong to the group of ultramafic lamprophyre and, more strictly, it should be called melnoite defined by Mitchell (1994).1) This finding should contribute to the understanding of the origin of carbonatite magma and the relationships between carbonatite and silicate magmas.

## Introduction

Some of lamprophyric rocks contain magmatic carbonate minerals. Although the genesis and evolution of lamprophyres are controversial,2) many researchers have observed close relationships between ultramafic lamprophyres (alnöites etc.) and carbonatite. Examples of ultramafic lamprophyre–carbonatite association are found in Alnö, Sweden,3) Kandalaksha, Russia,4) and Damaraland, Namibia5) etc.. The approaches from geology, petrology, mineralogy, geochemistry and experimental petrology for such rock suites may provide a good clue to account for a genetic connection between the rocks.

On the other hand, the genetic relationship between kimberlite and carbonatite are also actively discussed (e.g. Le Bas, 1977,6) Gasper *et al*., 19847)). Diamondiferous kimberlites, carbonatites and lamprophyres occur in western Shandong province, China. The kimberlite occurs in the central part of western Shandong platform whereas carbonatites and lamprophyres in the north and south marginal part of the platform. Such occurrences have attracted attention of many researchers to obtain genetically significant implication between the kimberlite and the carbonatite. 8),9) In western Shandong platform, the carbonatite–kimberlite association also has been observed. In the course of our carbonatite study in the Laiwu–Zibo area, the north marginal part of western Shandong platform, we found extremely carbonate-rich lamprophyric rock. The rock used to be described as carbonatite by Wan *et al*. (1983).10) However, the rock has rather high silicate contents and its name “carbonatite” is not appropriate. It should be emphasized that the carbonate of the rock is essentially dolomite and the rock occurs as a diatreme breccia. Such dolomitic lamprophyre diatreme breccia has not been reported so far. This paper briefly describes the occurrence, whole-rock major and trace element concentrations of the rock as well as the features of the constituent minerals.

## Geological setting

**Fig. 1:**
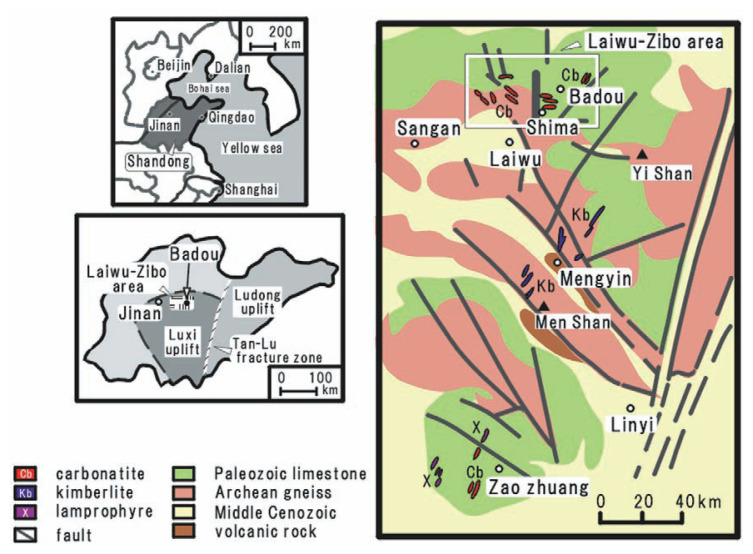
Location and geological maps of the Luxi uplift, Shandong province, China (modified from Wan *et al*., 1983).

The Laiwu–Zibo area where the dolomitic lamprophyre occurs locates in the northern margin of the Luxi uplift in Shandong province, China (Fig. 1). The Laiwu–Zibo area is geologically important field because nearly one hundred carbonatite and glim-merite bodies (dikes, sheets and rarely diatremes)10) distribute. In the central area of the Luxi uplift, some kimberlite bodies distribute. Cambrian and Ordovician limestone and Archean gneiss (Taishan group) distribute as basement rocks in the Luxi uplift. The basement is cut by many faults striking northwest11) and gigantic Tan–Lu fracture zone extending N-S direction bounds the eastern end of the Luxi uplift. Although the Laiwu–Zibo area is apart of the Luxi uplift, abundant carbonatite and glimmerite bodies appear in the former (Fig. 1).10) The carbonatitic rocks in this area contain high amount of silicate components, but they were classified into carbonatite. 9)

The dolomitic lamprophyre diatreme investigated is hereafter designated as Badou pipe after Wan *et al*. (1983).10) The pipe forms oval-shaped diatreme and its major axis is approximately 300 m long on the surface.10) Ordovician limestone surrounds the Badou pipe as the country rock. The pipe yields mica-rich brecciated carbonatitic rocks which have particularly high SiO_2_ contents among the other carbonatitic rocks in the Laiwu–Zibo area. The pipe brings a number of xenoliths from the depth of this area. Basalt, syenite and some sedimentary rocks have been found as xenoliths so far.

## Brief descriptions

**Fig. 2:**
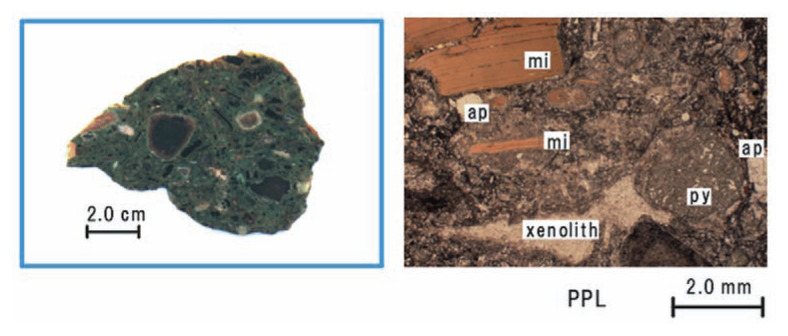
Sectioned sample and photomicrograph. Abbreviations: ph = phlogopite, ap = apatite, py = clinopyroxene, ca = carbonate mineral. PPL = plane–polarized light.

The rocks from Badou pipe are characterized by large phlogopite (up to 1.5 cm in length) and abundant dolomite. The rocks look dark green and clearly contrast with the surrounding limestone. On the hand specimen, many of xenoliths such as basalt, syenite, mudstone, limestone, marble, peridotite and quartzite can be perceivable (Fig. 2). The color of the xenoliths is various; they are black, white, orange, cream yellow or light green. Most of carbonate minerals are anhedral in matrix and the ratios of Ca to Ca+Mg are approximately 0.58–0.60, which are close to dolomite composition. Only small peaks of calcite were detected by XRD. Dolomite exceeds phlogopite in amount, while the amount of phlogopite is also large (ca. 6 volume%). Phlogopite is iron-rich (Mg/(Mg+Fe) = 0.72–0.80) and many crystals have deformation bands. Clinopyroxene, olivine, apatite, analcime, alkali-feldspar, amphibole, titanomagnetite and pyrite are included. Most of olivine has been serpentinized. The amount of each mineral is much less than that of phlogopite. Clinopyroxene is diopside and rimmed with carbonate and round. Apatite crystals are invariably euhedral; some grains are columnar and the others hexagonal. Although most of analcime and alkali-feldspar occur in the matrix as anhedral crystals, they may be primary phases. Amphibole appears only in reaction rims surrounding xenoliths.

## Analytical method

Major and trace element concentrations were obtained by XRF analysis using a Rigaku RIX 2100 XRF spectrometer at the Institute of Mineralogy, Petrology and Economic Geology, Tohoku University.

The samples were coarsely crushed and fragments of xenoliths were removed to the maximum extent. Each rock powder thus made was heated to 110 °C in an oven longer than four hours and subsequently ignited at approximately 1000 °C in a muffle furnace for long time (40 hours) to vaporize the large quantity of volatile component. During the ignition, all the H_2_O(+) and CO_2_ contained in the sample should have been eliminated. Glass beads for XRF analysis were made from this powder. Two gram of sample powder and four gram of the lithium borate flux were mixed and then heated at 1050 °C using a high-frequency bead sampler according to Goto *et al*. (2002).12)

The calibration curves were obtained by measuring the GSJ geochemical standards.12) An Rh/W dual X-ray tube was used in the XRF analysis. The accelerating voltage and electron current were set at 50 kV and 50 mA, respectively.

## Results and discussion

The whole-rock major and trace element compositions of the carbonatitic rocks from Badou pipe are presented in Table I. Note the very small variation of the measured elements: they must have consisted of a single body of melt, though negligible differences exist. The chemical compositions of the rocks from Badou pipe are very characteristic: they contain approximately 19.4 wt.% CaO, 7.7 wt.% MgO and 9.0 wt.% Fe_2_O_3_ * on averages. All the three components might have existed as parts of carbonate compounds. Their SiO_2_ and Al_2_O_3_ contents, however, are considerably high for the Badou rocks. Both SiO_2_ and Al_2_O_3_ are major silicate components, and therefore the samples must have contained too much silicate components to regard these rocks simply as carbonatite.

LOI ranges from 16.9 to 18.4 wt.% for the rocks from Badou pipe. Most of LOI are considered to have derived from CO_2_, though small quantities of F and Cl from apatite and S from pyrite may be contained in LOI. If a rock consists of half dolomite and half forsterite, then the rock should contain ca. 22 wt.% CO_2_. In the case of a rock consisting of half dolomite and half fayalite, it must have 19 wt.% CO_2_. The density of forsterite and fayarite used in the calculation range from 3.2 to 4.4 g/cm^3^. If the rocks from Badou pipe are genuine carbonatite, CO_2_ contents of the Badou rocks must exceed 19–22 wt.%. Therefore, even if all LOI is CO_2_ and has been derived from the dolomite decomposition, its measured amount is not enough. Although CO_2_ content of the Badou rocks is large, its quantity is less than the carbonatite of IUGS classification.13)

The chemical compositions of carbonatites reported so far and those of the rocks from Badou pipe are compared and shown in Fig. 3.14) SiO_2_ contents of the rocks from the pipe plot outside of the range of carbonatite. The abundances of CaO and MgO are within the carbonatite ranges, though the rock from the pipe shows minimal content. The rocks from Badou pipe are characterized by higher SiO_2_ content and less CO_2_ than common carbonatite. These chemical data strongly suggest that the rocks from Badou pipe are not classified into carbonatite. Accepting IUGS recommendation,13) we have to put some prefix like calcitic or carbonatitic in front of the exact rock name.

**Fig. 4:**
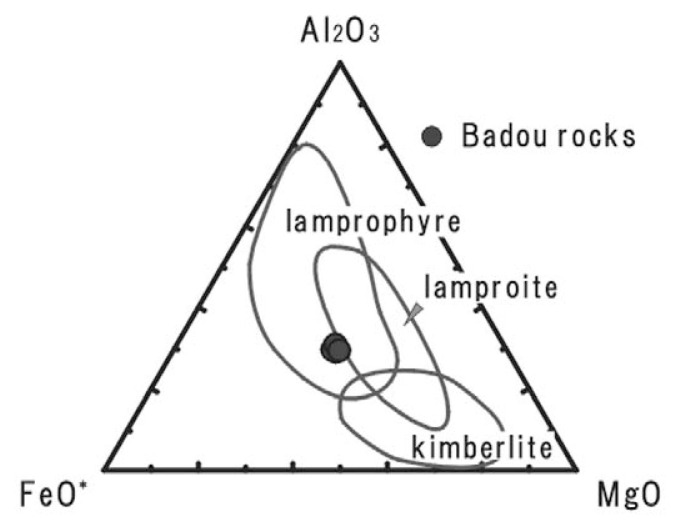
MgO–Al_2_O_3_–FeO* diagram indicating the compositional fields of lamprophyre, lamproite and kimberlite. From Bergman (1987).

**Fig. 5:**
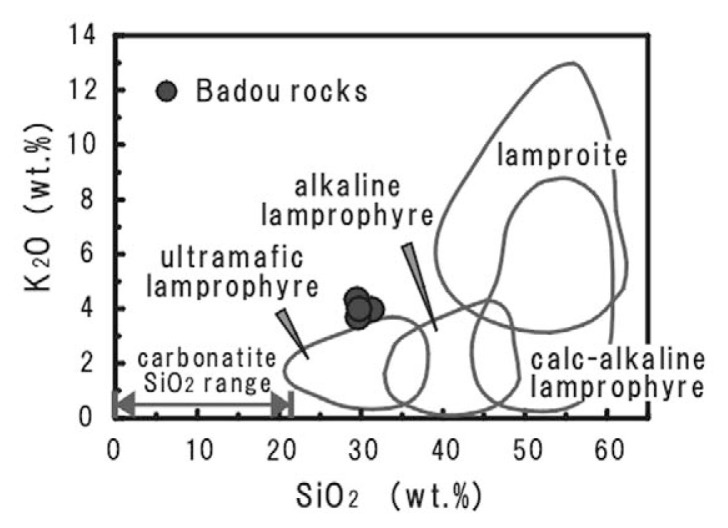
K_2_O versus SiO_2_ plot. The fields of each rock type are reproduced from Barbieri *et al*. (1997) and the concentration range for carbonatites are from Woolley *et al*. (1989).

**Fig. 6:**
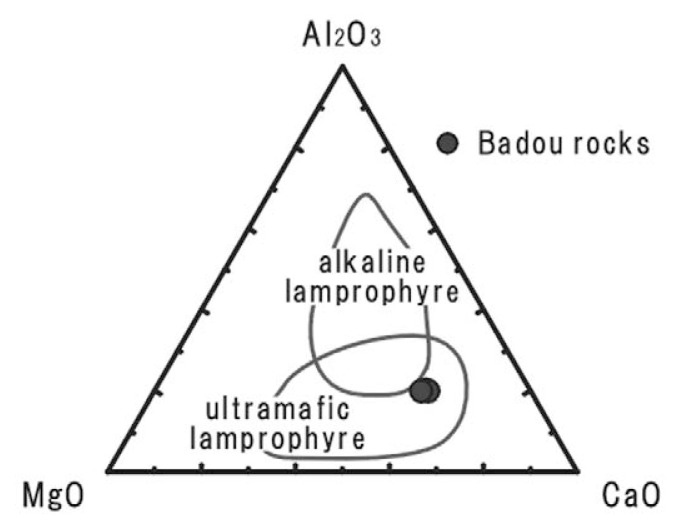
CaO–Al_2_O_3_–MgO plots discriminating the compositional fields of alkaline lamprophyre and ultramafic lamprophyre. Data from Barbieri *et al*. (1997).

The rock specimens contain large amounts of silicate minerals and SiO_2_ is the most dominant; Al_2_O_3_ is major component too. Thus the rock seems to be a kind of unusual silicate rocks, and we will examine what the most adequate name is. The SiO_2_ concentrations of the rocks from the pipe are similar to the least SiO_2_ contents of ultramafic silicate rocks. The rocks from Badou pipe fall between carbonatite and ultramafic silicate rocks. We therefore tried to compare the rocks from Badou pipe with some ultramafic silicate rocks and discuss the nature of the Badou rocks. The MgO–Al_2_O_3_–FeO* ternary diagram (Fig. 4) is employed for distinguishing ultramafic silicate rocks; i.e., lamprophyre, lamproite and kimberlite.15) The rocks from Badou pipe fall inside of lamprophyre field and plot outside the boundary of lamproite field. None of them plot in the kimberlite field. The discrimination may conclude that the rocks of Badou pipe belong to lamprophyre group. The difference of the whole-rock compositions among ultramafic lamprophyre, alkaline lamprophyre, calc-alkaline lamprophyre and lamproite are shown in the K_2_O versus SiO_2_ diagram (Fig. 5).14),16) The rocks from Badou pipe have lower SiO_2_ and K_2_O contents than lamproites. They also plot closely to the field of ultramafic lamprophyre, though they show slightly higher K_2_O contents than ultramafic lamprophyre. The CaO–Al_2_O_3_–MgO ternary diagram (Fig. 6) is used to discriminate ultramafic lamprophyre from alkaline lamprophyre.16) The rocks from Badou pipe plot in the field of ultramafic lamprophyre, but not in the alkaline lamprophyre field in Fig. 6. The concentrations of Al_2_O_3_ and CO_2_ have a definite influence to the final decision. Those element abundances differ much between the ultramafic lamprophyre and the alkaline lamprophyre. The Al_2_O_3_ contents are statistically 13.7 ± 2.7 wt.% for alkaline lamprophyre and 6.8 ± 3.0 wt.% for ultramafic lamprophyre.18) Approximately 6.7 wt.% of Al_2_O_3_ content in the Badou rocks is in good agreement with the ultramafic lamprophyre. Moreover, whole-rock CO_2_ contents of these rocks estimated from the LOI (LOI = 16.9–18.4 wt.%) are also in accord with the view that the rock from Badou pipe is ultramafic lamprophyre. CO_2_ content of ultramafic lamprophyre is variable and much higher (8.2 ± 6.9 wt.%) than that of alkaline lamprophyre (2.7 ± 2.4 wt.%).17) Consequently, the Badou rocks are ultramafic lamprophyres.

Let us proceed to further classify the ultramafic lamprophyres since they are so diverse group of rocks and the definition is very complicated.2) Mitchell (1994)1) proposed that any ultramafic lamprophyres closely related with carbonatitic melt should be named as melnoite. The mineral assemblage of the rocks from Badou pipe resembles that of melnoite.1) The main mineral phases such as carbonate, phlogopite, olivine, clinopyroxene and analcime correspond to the mineral assemblage of melnoite compiled by Mitchell (1996).18) Notice that the reported analytical results of the averaged carbonate melnoites in Table I19): the chemical compositions of the rocks from Badou pipe are very close to the reported average. Therefore, these data confirm that the Badou rocks belong to melnoite. It is also important to emphasize that the melnoite from Badou pipe contains a large amount of dolomite. In contrast, calcite is not so abundant. The dolomitic melnoite is a befitted term for the rock from Badou pipe.

**Fig. 7:**
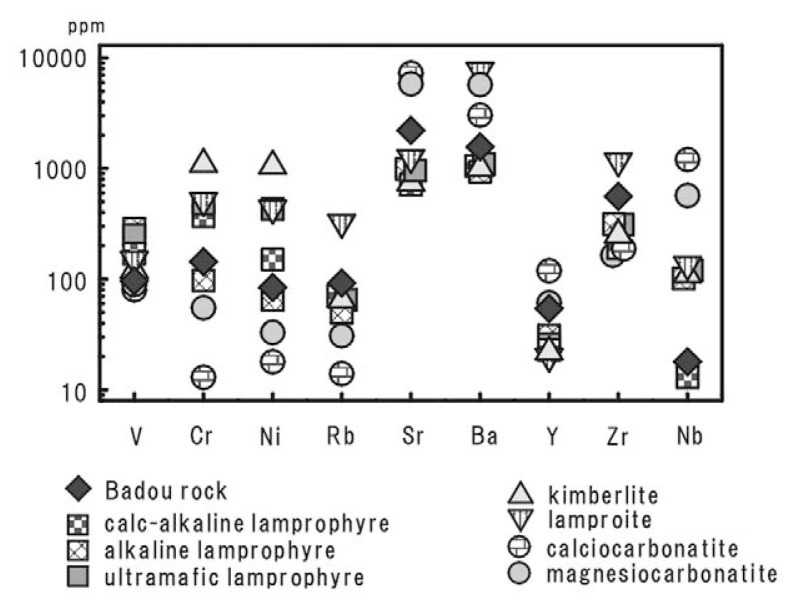
Comparison of averages of trace element concentrations in the rocks from Badou pipe with the ultramafic silicate rocks and two different carbonatite types. Data for the ultramafic silicate rocks are modified from Rock (1991) and carbonatites are applied to Woolley (1989).

The compositions of trace elements of the Badou melnoite are presented in Table I. Their features are compared to the average of five ultramafic silicate rocks,2) and two carbonatite types, i.e., calciocarbonatite and magnesiocarbonatite14) (Fig. 7).

Cr and Ni concentrations of the melnoite from Badou pipe are nearly one order of magnitude lower than those of kimberlite and lamproite and more enriched in Cr and Ni than calciocarbonatite and magnesiocarbonatite. Y and V contents of the Badou melnoite are nearly the same as those of magnesiocarbonatite. As far as Y and V abundances are concerned, the Badou rocks have carbonatitic characters, but not the affinities with the ultramafic silicate rocks (Fig. 7). In contrast, the concentrations of Sr, Ba, and Nb of the Badou melnoite are different from those of calciocarbonatite and magnesiocarbonatite. The Badou melnoite show depletion in those elements as compared with the carbonatites. Especially Nb content of the Badou melnoite (approximately 20 ppm) is very low. The overall feature of the trace-element concentrations is that the Badou melnoite lies between ultramafic lamprophyre and magnesiocarbonatite. This relationship is clearly seen in Cr, Ni, Sr and Ba concentrations. The Badou rock has also some affinity to alkaline lamprophyre. Thus the trace element characteristics of the Badou melnoite clarified that the rocks have both lamprophyric and carbonatitic characteristics.

Many ordinary carbonatite bodies and some unique carbonatitic rocks appear in the Laiwu–Zibo area. We can find the variety of rocks in the Laiwu–Zibo area: one of the significant rock association is melnoite– carbonatite–glimmerite. Although lamprophyre–carbonatite and carbonatite–glimmerite with or without alkaline rock associations are rather common, we report the first discovery of the special rock association from the Laiwu–Zibo area. All the three special kinds of rocks erupted or intruded in the Laiwu–Zibo area within a similar time and limited place. A number of carbonatite and glimmerite appear as dikes and sills close to Badou in the Laiwu–Zibo area.9) Similar rock associations are reported from Kandalaksha in Russia20),21) and from Norseman in western Australia.19) The intra-montane ultra-alkaline province in central Italy 22) may also be the typical area where a similar melnoite–carbonatite association appears. However, melnoite–carbonatite–glimmerite association appears in none of the above-mentioned localities.

The ultramafic lamprophyres and carbonatites occur in the islands of Kandalaksha gulf, near Kola peninsular, as intrusions. The ultramafic lamprophyres are mostly fine-grained porphyritic rocks.20) The mineral assemblages of the lamprophyres from the islands bear some resemblance to the Badou dolomitic melnoite but the former contains ankerite, Fe-rich dolomite, and also garnet and aegirine set in the groundmass in addition to the commonly recognized minerals in the Badou melnoite. The carbonatites in the islands of Kandalaksha gulf are ferrocarbonatite. The carbonate mineral of the Badou melnoite is mainly dolomite, and a few carbonatite in the Laiwu–Zibo area are classified into magnesiocarbonatite because of high Mg content.

The Norseman melnoites, reported from western Australia, occur in the southeastern margin of the Yilgarn Craton as dikes. Graham *et al*. (2002)19) classified them as clinopyroxene–phlogopite melnoite and they are hypabyssal. The phenocrysts assemblage of the Norseman melnoites is olivine, phlogopite, ilmenite and clinopyroxene. In addition to these minerals, calcite, magnetite and perovskite can be recognized in the groundmass. The Badou melnoite, however, differs from the Norseman melnoite; the former contains abundant dolomite. Small differences of chemical compositions can be noticed between the Badou and Norseman rocks, but the composition of the Badou melnoite closely resembles the carbonate melnoite reported by Graham *et al*. (2002)19) (Table I). The difference of chemical compositions between Norseman melnoite and Badou melnoite may be affected by the amount of dolomite component.

A few diatremes and tuff rings, which melilitite or carbonatite consist of, are reported from four areas in central Italy. According to Mitchell (1996),18) both melilitite and melnoite belong to melilitite clan, although their facies are different. We recognized that the association of the igneous products in the Laiwu–Zibo area is similar to that of central Italy where carbonatite and ultramafic silicate rocks coexist. It should be pointed out that some diatremes occur in these areas as well.22) Stoppa *et al*. (1997)22) identified two monticellite calciocarbonatite diatremes in Polino. Although the rocks from one of the diatremes are nearly the same as that of the Badou melnoite, the quantity of carbonate minerals in Polino monticellite calciocarbonatite is more than that in the Badou melnoite. The minerals in tuffistic carbonatite from Polino are characterized by phlogopite, olivine, Th–perovskite, Ti–magnetite and Zr–schorlomite with commonly occurring minerals in carbonatitic rocks. Both the Polino carbonatite and Badou melnoite include a lot of phlogopite and olivine in common. The carbonate minerals, however, differ significantly between them. Main carbonate is calcite in Polino monticellite calciocarbonatite but dolomite in the Badou melnoite.

The Laiwu–Zibo area is an important locality to investigate the generation of carbonatitic rocks and the genetical relationships between melnoite, carbonatite and glimmerite. The magnesian carbonate-rich melts are generated by melting of carbonated peridotite at depth to 2–3.5 GPa.23) The magnesian carbonate-rich melt could be one of the candidate components for the melnoite of Badou pipe. The melnoite–carbonatite association can be explained by successive eruption during immiscible separation between silicate and carbonate components.22) The alkali components might have been procured from athenospheric mantle when the presumed plate collided in western Australia.19) A similar event might have happened when Sino–Korean and Pacific plate collided and the alkali-rich melt in the athenosphere would have generated beneath the Shandong area and glimmerite could be formed. We, however, point out that the magnesian carbonate-rich melts might have been parents of the glimmerite; the latter could be derived from silicate–carbonate mixed melt by magmatic differentiation; i.e. either by liquid immiscibility or crystal fractionation.

## Conclusion

The dolomitic melnoite has been found for the first from Badou in the Laiwu–Zibo area, China and its occurrence is unexpectedly a diatreme. The rock suite of this area consists of melnoite–carbonatite–glimmerite; such association has not been reported yet so far. The melnoite has high carbonate component and a large amount of Fe-rich phlogopite. The Badou melnoite includes not only phlogopite but a number of phenocrysts of diopside, olivine and apatite as well, and supplies a large amount of diverse xenoliths. Although melnoite belongs to ultramafic silicate rock, the Badou melnoite melt must have contained carbonate melt nearly one third of the total weight. The concentrations of some trace elements are similar to those of the ultramafic silicate rocks, and the other to carbonatitic rocks.

## Figures and Tables

**Fig. 3:**
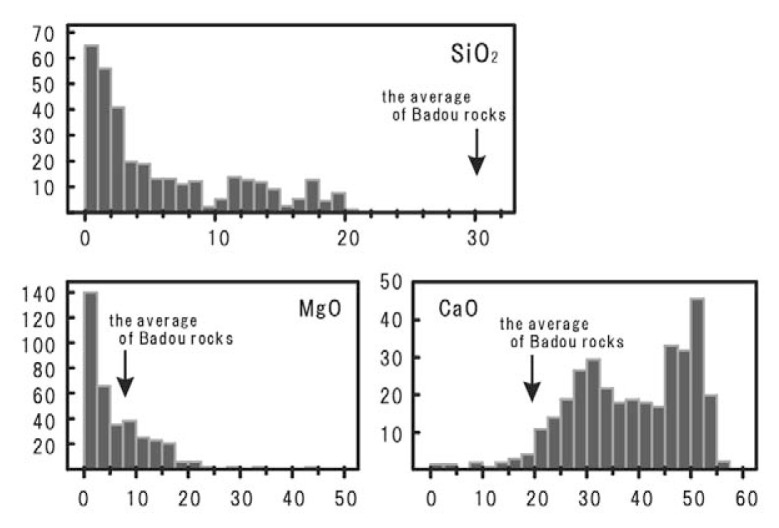
Frequency distribution diagrams of SiO_2_, MgO and CaO for acceptable carbonatite (refer to Woolley *et al*., 1989) and the average of the rocks from Badou pipe.

**Table I:** Major and trace element compositions for the rocks from Badou pipe

	CaM-G1	CaM-F5	CaM-F6	CaM-F7	carbonate+ melnoite
wt%					
SiO_2_	30.99	29.79	29.87	29.98	29.5
TiO_2_	0.91	0.91	0.94	0.91	3.4
Al_2_O_3_	6.87	6.61	6.76	6.63	5.4
Fe_2_O_3_*	8.93	9.02	9.05	9.07	16.4
MnO	0.11	0.11	0.11	0.11	0.25
MgO	7.59	7.50	7.93	7.74	15.9
CaO	19.30	19.48	19.53	19.31	13.5
Na_2_O	2.10	1.89	2.03	2.05	0.45
K_2_O	3.99	4.23	3.80	4.00	1.6
P_2_O_5_	3.03	2.96	3.10	3.11	0.82
H_2_O(−)	0.43	0.37	0.52	0.48	–
LOI	16.87	18.42	17.37	17.78	14.5
Total	101.11	101.28	101.01	101.16	101.7

ppm					
V	99	95	96	96	–
Cr	143	124	156	150	699
Ni	84	78	88	85	389
Rb	93	91	90	92	–
Sr	2188	2335	2100	2232	–
Ba	1497	1655	1618	1505	1487
Y	53	54	55	54	32
Zr	555	555	563	564	245
Nb	17	21	17	18	137

Fe_2_O_3_*: total Fe as Fe_2_O_3_. LOI: Loss on ignition.

+ carbonate melnoite from Graham *et al*. (2002).

−: not reported.
